# Use of Dynamic Ultrasonography in the Evaluation of Meniscal Extrusion: A Technical Report

**DOI:** 10.7759/cureus.93539

**Published:** 2025-09-30

**Authors:** Arifur Rahman, Julio Fernandes, Tarek Boutefnouchet

**Affiliations:** 1 Trauma and Orthopaedics, University Hospitals Birmingham NHS Foundation Trust, Birmingham, GBR

**Keywords:** clinical assessment tools, dynamic, dynamic ultrasonography, extrusion, meniscal

## Abstract

Meniscal root tears can lead to extrusion with biomechanical consequences similar to total meniscectomy. This report describes a protocol for bedside ultrasonography (real-time ultrasound imaging at the point of care) for dynamic assessment of meniscal displacement before and after surgery. Using a 5-12 MHz linear probe, the meniscus is evaluated under stress and motion to assess reducibility and repair integrity. Unlike MRI, this method offers real-time, load-responsive imaging. Dynamic ultrasound serves as a practical adjunct to MRI, aiding surgical planning and improving postoperative assessment in patients with meniscal root injuries.

## Introduction

Meniscal root tears are now recognized as more common than previously thought [1]. In biomechanical terms, an extruded meniscus with loss of root attachment is considered functionally equivalent to a total meniscectomy [1,2]. Meniscal extrusion refers to the displacement of the meniscus beyond the peripheral margins of the tibial plateau, typically defined as greater than 3 mm on coronal imaging [3,4]. An extruded meniscus can lead to altered load distribution in the knee, accelerating cartilage degeneration and contributing to early osteoarthritis and persistent knee pain [5].

This association is well established: multiple MRI-based cohort studies have demonstrated that medial meniscal extrusion predicts accelerated cartilage loss, increased pain, and structural progression of osteoarthritis, a finding further confirmed by a 2024 systematic review encompassing 19 clinical studies [6,7]. The medial meniscus is more frequently affected than the lateral meniscus due to its association with posterior root tears and firmer capsular attachments [4].

Magnetic resonance imaging (MRI) remains the gold standard for assessing meniscal pathology due to its excellent soft tissue contrast and anatomical detail [2,3]. However, MRI is a static modality, which limits its ability to evaluate dynamic meniscal displacement under physiological load [8]. Ultrasonography, in contrast, is radiation-free, cost-effective, and accessible, allowing real-time, weight-bearing imaging of the meniscus. Studies have demonstrated that ultrasound can detect occult extrusion not visible on MRI, providing additional insight into meniscal function [5,8,9].

This is a technical and descriptive report rather than a hypothesis-driven or validation study. Its goal is to present a step-by-step, reproducible ultrasound protocol for assessing meniscal extrusion dynamically, illustrating how the technique can complement MRI in both preoperative assessment and postoperative follow-up.

## Technical report

Equipment

The equipment needed is: (i) Ultrasound machine and (ii) High-frequency linear probe (5-12 MHz)

Patient preparation and positioning

The following steps should be followed: (i) Obtain consent and expose the patient from mid-thigh to mid-tibia; (ii) The patient should lie supine on the operating table, knee extended and supported by a small bolster under the popliteal fossa to ensure the posterior capsule (back lining of the joint) is relaxed; (iii) For assessment of weight-bearing mechanics, the authors perform scans with the patient sitting upright, feet flat on the floor-allowing varus/valgus movements (inward/outward angulation of the knee).

Scanning protocol

The protocol to be followed while scanning is as follows: (i) Landmarks: Palpate the medial joint line between the medial femoral condyle and tibial plateau; (ii) Longitudinal Orientation: The probe is aligned parallel to the medial joint line, just posterior to the tibial plateau margin; (iii) Identification of Meniscus: The triangular meniscal body appears as a homogeneous, hyperechoic (bright on ultrasound) structure. The free edge and peripheral capsular attachments are traced from the anterior horn to the posterior root; (iv) Extrusion Measurement: Using electronic calipers, the distance from the outermost echogenic (bright) line of the meniscus to the edge of the tibial plateau is recorded at full extension. Values ≥3 mm define significant extrusion; (v) Transverse Views: A transverse sweep verifies horn morphology and excludes radial tears (vertical cleavage tears) that may change extrusion patterns.

Dynamic evaluation

**Figure 1:**
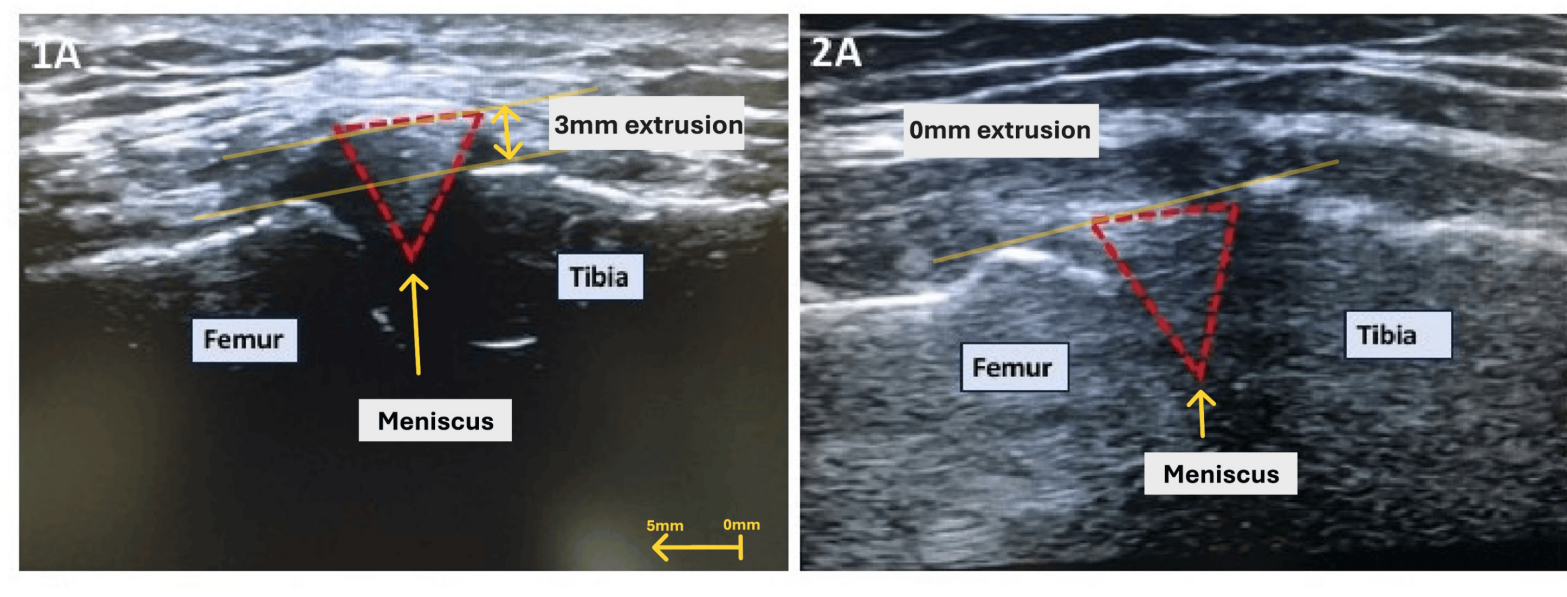
Static ultrasound image of a medial meniscus showing an extruded meniscus by 3 mm when the compartment is loaded (A) and an image taken two weeks postoperatively following root repair showing a reduced and stable meniscus (B). Image Source: Tarek Boutefnouchet

The examiner applies gentle varus and valgus stress (manual inward/outward pressure) or instructs the patient to shift weight medially or laterally. The probe remains over the joint line: (i) Varus/Valgus Stress (Supine): At 0°, 30°, 60°, and 90° flexion; (ii) Seated Weight-Shift: Medial and lateral load simulation; (iii) Flexion Sweep: Incremental flexion from 0° to 90°. This can be repeated postoperatively to assess for stability of the meniscal root (Figure 1).

For patients unable to safely tolerate full standing, seated scanning with feet flat on the floor serves as a partial weight-bearing simulation. Although not equivalent to full axial loading, even partial weight-bearing has been shown to increase tibiofemoral (thigh-shin joint) contact forces, an effect sufficient to provoke clinically meaningful meniscal extrusion in cases of root insufficiency. This approach, therefore, represents a safer and more reproducible alternative for early postoperative or symptomatic patients [7].

## Discussion

A key indicator of successful meniscal root repair is the reduction of meniscal extrusion, as persistent extrusion is associated with ongoing loss of hoop stress and progression of osteoarthritis [5]. Studies demonstrate that meniscal root tears lead to significant biomechanical consequences, such as a reduction in tibiofemoral contact area and a substantial increase in joint contact pressures [1,2,10]. Root repairs that fail to restore normal joint biomechanics are associated with persistent meniscal subluxation and inferior clinical outcomes [11].

**Figure 2:**
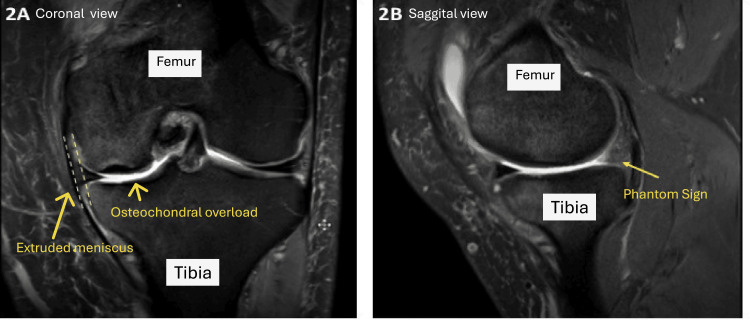
T2 MRI image of the knee demonstrating an extruded meniscus associated with meniscal root tear is characteristic of root tear (A); the lesion demonstrates the so-called pathognomonic Phantom sign with loss of meniscal contour on the image (B). Note how the patient shows associated signs of osteochondral overload on the medial femoral condyle. Image Source: Tarek Boutefnouchet

Recent research highlights the importance of evaluating meniscal extrusion both preoperatively and postoperatively to assess surgical outcomes [8,9]. Traditionally, MRI has been considered the gold standard for diagnosing meniscal root tears and evaluating meniscal extrusion, given its excellent soft tissue contrast and detailed anatomical visualization (Figure 2) [3]. However, MRI remains a static modality and cannot assess the dynamic nature of the meniscus under physiological loading [8].

Ultrasound offers several advantages: it is safe, inexpensive, widely accessible, and enables real-time dynamic assessment of meniscal movement under various loading conditions, although operator dependent [5,8]. Studies have demonstrated that ultrasound can detect meniscal extrusion not visible on static MRI, particularly under weight-bearing conditions, and may correlate more closely with symptoms such as pain [8]. Moreover, meniscal extrusion that is reducible under dynamic loading conditions is more likely to be restored by root repair, whereas fixed extrusion may suggest chronic degeneration and predict less favorable outcomes [9].

Overall, the use of dynamic ultrasound represents a promising advancement in the management of meniscal root tears and meniscal extrusion. Future research should focus on validating this protocol by assessing inter-observer reliability, correlating findings with MRI, and defining quantitative thresholds for dynamic extrusion, which lie beyond the scope of this descriptive report.

## Conclusions

Bedside dynamic ultrasonography is a simple and accessible technique that can visualise meniscal extrusion in real time. By assessing meniscal mobility under physiological load, it offers complementary information to static MRI and may support surgical planning and postoperative monitoring in patients with meniscal root tears. While this protocol demonstrates feasibility and potential utility, further studies are needed to validate its reproducibility, define dynamic thresholds, and establish correlations with clinical outcomes.
